# Extraosseous Spread of Multiple Myeloma Mimicking Pancoast Tumor

**DOI:** 10.7759/cureus.22412

**Published:** 2022-02-20

**Authors:** Kuldeep Bansal, Sumedha Singh, Anuj Gupta, Harvinder Singh Chhabra, Kalyan Kumar Varma Kalidindi

**Affiliations:** 1 Orthopedics, Indian Spinal Injuries Center, Delhi, IND; 2 Radiology, Instiute of Medical Sciences and SUM Hospital, Siksha 'O' Anusandhan University, Bhubaneswar, IND

**Keywords:** bone marrow, plasma cell, pancoast tumour, extra-osseous, multiple myeloma

## Abstract

A 55-year-old man presented with upper backache for one month, inability to move both the lower limbs for two weeks and retention of urine for five days. Examination revealed spastic paraplegia and reduced breath sounds in the right upper zone. Initial imaging revealed a soft tissue lesion in the apex of the right lung, suggesting a Pancoast tumor. It also showed a lytic, expansile lesion of the T2 vertebra extending to the right second posterior rib on subsequent imaging. High serum calcium, M-spike in beta-gamma globulin region on serum electrophoresis, 50%-60% plasmacytoid cells on bone marrow aspiration, concertina collapse of the vertebral body, and pattern of neurological deficit pointed towards multiple myeloma. T2 corpectomy and mesh cage placement, C7-T4 posterior stabilization, and resection of the second rib were done. Histopathology confirmed multiple myeloma. Postoperatively, the patient was managed with radiotherapy and bortezomib. The patient had a good neurological recovery. Timely intervention is critical for disease control and leads to better recovery.

## Introduction

Multiple myeloma can present with a pathological vertebral fracture with or without the neurological deficit and characteristic blood picture [1]. Extraosseous involvement is found in less than 10% of multiple myelomas, and its presence is usually associated with poorer outcomes [2]. Pancoast tumors are usually malignant neoplasms of the lung located at the apex on the superior sulcus [3]. We report a challenging case of cord compression secondary to extraosseous spread of multiple myeloma, mimicking a Pancoast tumor initially due to its location. Such a case has not been reported previously.

## Case presentation

A 55-year-old man presented with upper backache for one month, inability to move both the lower limbs for two weeks and retention of urine for five days. It was not associated with fever, weight loss, trauma, cough, or hemoptysis. The patient was a chronic smoker with a history of 30 pack years. Neurological examination revealed grade 0 power (Medical Research Council, MRC) and grade I spasticity (Modified Ashworth Scale) in both lower limbs. Both knee jerks were brisk. Bilateral ankle clonus and extensor plantar response were elicited. Sensations to pain and temperature were decreased below the T4 level. Deep tenderness was present at the T2-T3 midline and right paraspinal regions. The peri-anal sensation was intact, but the voluntary anal contraction was weak. General examination revealed reduced breath sounds in the right upper zone of the chest. The liver and spleen were not palpable. A provisional diagnosis of extramedullary compressive thoracic myelopathy was made.

**Figure 1:**
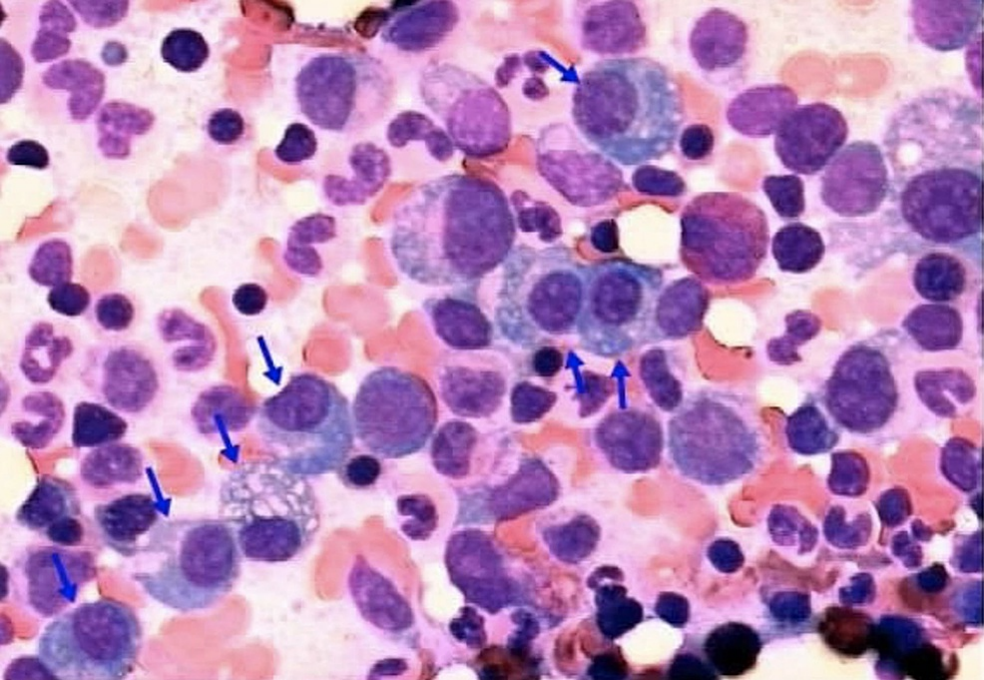
Bone marrow examination showed the presence of atypical plasma cells.

**Figure 2:**
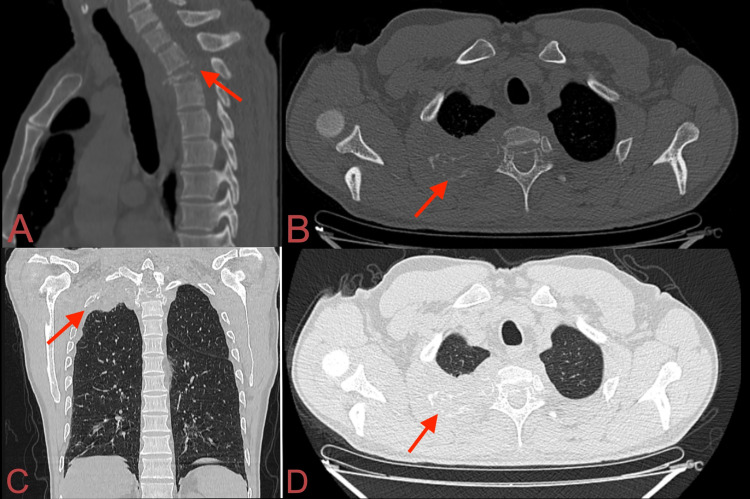
NCCT of the spine and chest. (A) Sagittal view in the bone window showed the collapse of the T2 vertebra with segmental kyphosis, (B) axial view in bone window lytic, expansile lesion of the T2 vertebra involving its body, right pedicle, right costotransverse joint, extending to the right second posterior rib, (C, D) coronal and axial views in lung window revealed a soft tissue lesion in the apical segment of right upper lobe, resembling a Pancoast tumour. NCCT: non-contrast computerized tomography

**Figure 3:**
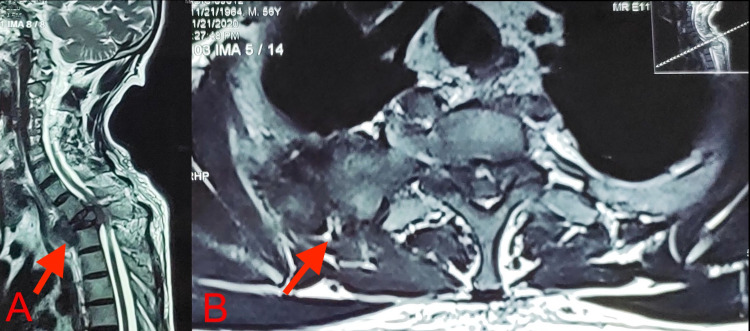
T2-weighted sagittal (A) and axial (B) MRI revealed the collapse of the T2 vertebral body and adjacent hypointense soft tissue component extending to the epidural space compressing the cord and paravertebral region creeping along the right second posterior rib. MRI: Magnetic Resonance Imaging

**Figure 4:**
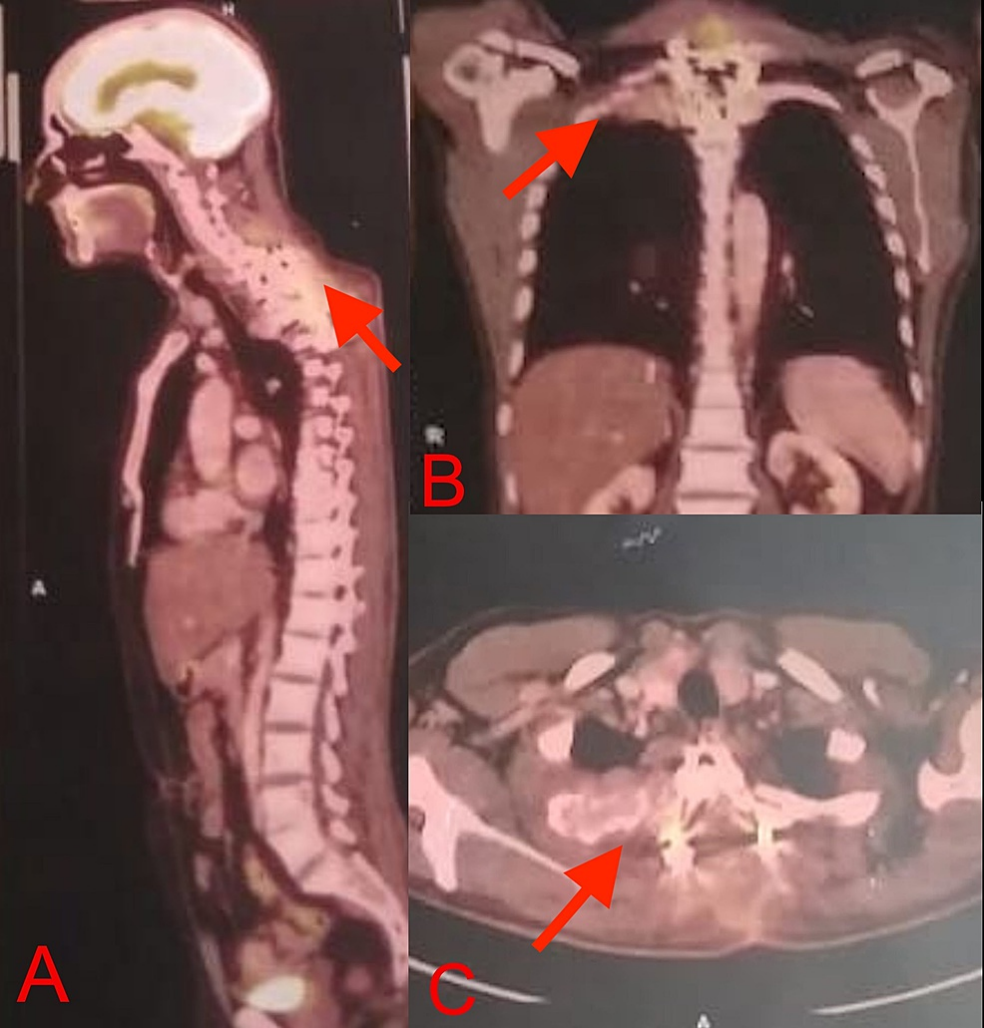
18F-FDG PET scan sagittal (A), coronal (B), and axial (C) sections showed isolated, focal increased uptake at the T2 vertebral level. 18F-FDG PET: fluorodeoxyglucose positron emission tomography

Laboratory tests revealed hemoglobin of 11 g/dl, white blood cell (WBC) count of 6540/mm^3^, erythrocyte sedimentation rate (ESR) of 15 mm/h, C-reactive protein (CRP) of 23.31 mg/L, serum calcium of 10.5 mg/dL, serum alkaline phosphatase (ALP) of 101 IU, and normal renal function tests. Serum electrophoresis revealed M-spike in the beta-gamma globulin region. Urine examination revealed no evidence of Bence Jones proteins. Bone marrow aspiration revealed 50%-60% plasmacytoid cells (normal 2%-3%) (Figure 1). Blood beta-2 microglobulin level was 2.6 mcg/mL (normal 0.7-1.8 mcg/mL). A plain radiograph revealed a concertina (concentric) collapse of the T2 vertebra with mild kyphosis. Non-contrast computerized tomography (NCCT) of the spine and chest revealed a lytic, expansile lesion of the T2 vertebral body and destruction of the second rib with the soft tissue component involving the apex of the right lung resembling a Pancoast tumor (Figure 2). Magnetic Resonance Imaging (MRI) revealed destruction of T2 vertebral body and right pedicle with hypointense paravertebral and epidural soft tissue component compressing the cord and creeping along the right second posterior rib (Figure 3). Fluorodeoxyglucose positron emission tomography (18F-FDG PET) showed isolated, focal increased uptake at T2 vertebral level (Figure 4).

**Figure 5:**
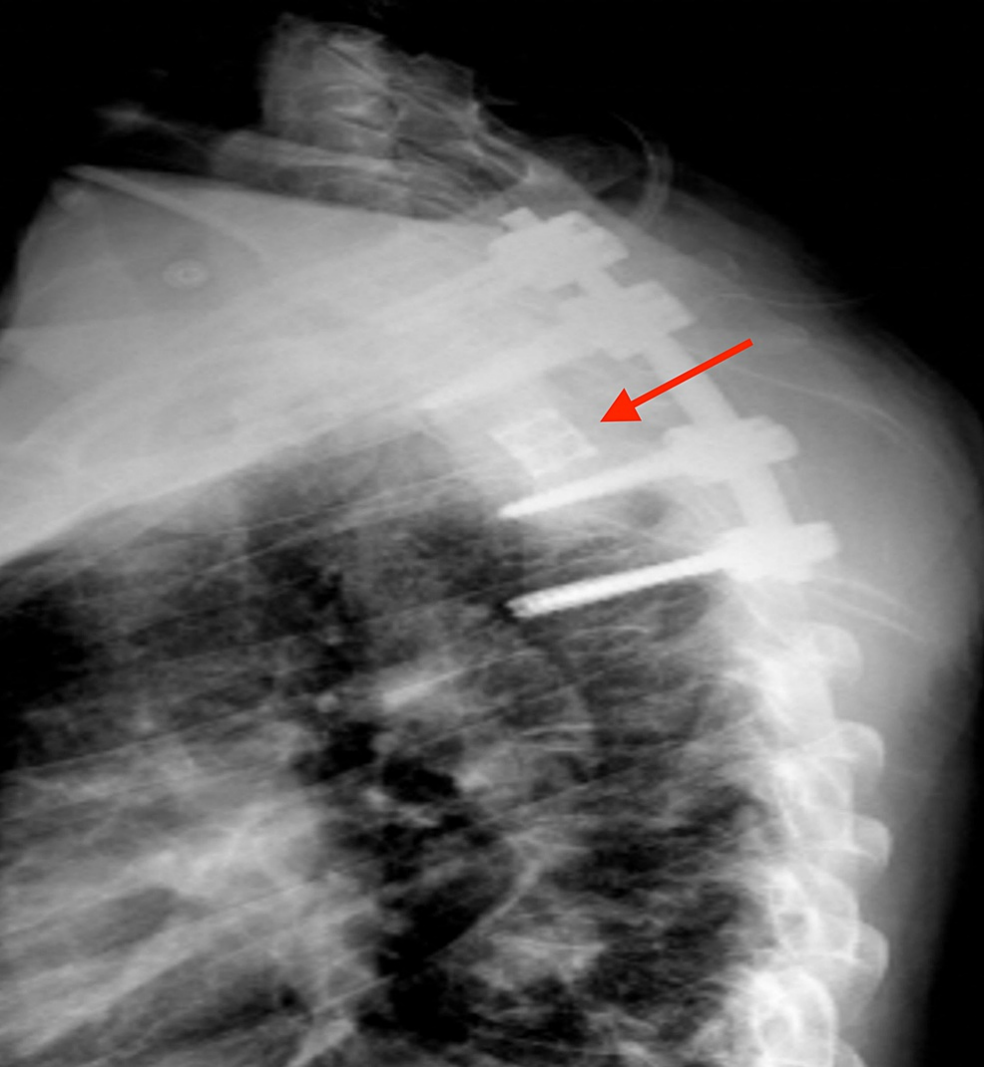
A postoperative lateral radiograph showed bilateral pedicle screw instrumentation from C7 to T4, T2 corpectomy, and placement of interbody mesh cage.

After informed consent, the patient was operated on with T2 corpectomy and mesh cage placement, C7-T4 posterior stabilization, and resection of the second rib (Figure 5). Bone and soft tissue histopathology revealed 50%-60% plasma cells, consistent with multiple myeloma. Rehabilitation and gradual back strengthening exercises were started from postoperative day two. The patient was initiated on radiotherapy (3000 cGy), injection bortezomib (1.3 mg/m^2^ intravenously followed by subcutaneous administration on days one, four, eight, and 11), lenalidomide (25 mg per orally daily on days 1-14), and dexamethasone (40 mg per orally daily on days one, eight, and 15). The patient improved from American Spinal Injury Association (ASIA) grade B to D over four to six weeks. At the latest follow-up of six months, the patient was doing well and had no new complaints.

## Discussion

Our case is an unusual presentation of the extraosseous spread of multiple myeloma that caused cord compression and resembled a Pancoast tumor, posing an initial diagnostic dilemma. Involvement of the apical segment of the right upper lobe of the lung and the second rib along with the T2 vertebra on imaging indicated towards Pancoast tumor. However, Pancoast tumor (superior sulcus tumor) typically arises from the groove superior to the first rib and presents with symptoms of ipsilateral arm pain, paresthesias, atrophy of intrinsic muscles of the hand, and Horner’s syndrome (ptosis, miosis, and anhidrosis) due to the involvement of the brachial plexus, sympathetic trunk, and stellate ganglion [4]. This patient, in contrast, had no symptoms of Horner’s syndrome or brachial plexus involvement. Presence of serum calcium towards the higher side of the normal range, M-spike in beta-gamma globulin region on serum electrophoresis, 50%-60% plasmacytoid cells on bone marrow examination, concertina collapse of the T2 vertebral body, and pattern of neurological deficit suggested multiple myeloma. Extraosseous extension of multiple myeloma is reported in <10% of patients [2].

Multiple myeloma is one of the commonest tumors of the bone [5]. It is characterized by malignant proliferation of plasma cells derived from a single B-cell lineage with suppression of normal humoral immunity. Widespread lytic lesions, recurrent infections, renal failure, and hypercalcemia are commonly associated. It is diagnosed with laboratory investigations, imaging, and bone marrow aspiration and biopsy [6]. 

Whereas multiple myeloma is a B-cell-derived malignant plasma cell tumor that spreads by the hematogenous route through the invasion-metastasis cascade [7], Pancoast tumor is typically a squamous cell carcinoma (non-small cell carcinoma variety) that spreads locally [8]. Besides initial laboratory investigations like kidney function tests and serum electrophoresis, confirmatory diagnosis of multiple myeloma can be made by demonstration of atypical plasma cells in bone marrow aspiration and biopsy [9]. On the contrary, there are no specific laboratory investigations for Pancoast tumors. Chest X-ray, as the initial imaging modality, can show obliteration of the apical segment of the upper lobe of the lung. CT scan of the chest can further help to define the extent of the tumor, satellite nodules, and mediastinal lymphadenopathy-findings that are vital in the staging of Pancoast tumor. MRI helps in better delineation of soft tissue involvement and assessment of the invasion of brachial plexus, vasculature, and spinal cord, and hence, is the imaging modality of choice [10]. Pancoast tumor carries a better prognosis if detected early [11]. On the other hand, patients with multiple myeloma with the extraosseous spread at the time of diagnosis or during treatment generally have poor outcomes [12]. 

Our patient had a good neurological recovery and improved from ASIA grade B to D over four to six months. In contrast, Ha et al. demonstrated three patients of multiple myeloma with cord compression who had poor neurological recovery [13]. There is no consensus in the literature regarding the management of multiple myeloma with extraosseous spread. In our patient, surgical stabilization with debulking followed by radiotherapy and chemotherapy yielded an excellent outcome. Hence, timely intervention is critical for disease control and leads to better recovery [14].

## Conclusions

Extraosseous spread of multiple myeloma is seen in less than 10% of cases and can mimic Pancoast tumor when in the upper thoracic region. However, laboratory findings, bone marrow examination, and biopsy from the lesion can confirm the diagnosis of multiple myeloma. Timely diagnosis and surgical stabilization with debulking followed by radiotherapy and chemotherapy can lead to favorable outcomes in cases of cord compression due to the extraosseous spread of multiple myeloma.
